# Hybrid Nanostructures Obtained by Transport and Condensation of Tungsten Oxide Vapours onto CNW Templates

**DOI:** 10.3390/nano11040835

**Published:** 2021-03-24

**Authors:** Lavinia Gabriela Carpen, Tomy Acsente, Veronica Sătulu, Elena Matei, Sorin Vizireanu, Bogdan Ionuț Biță, Gheorghe Dinescu

**Affiliations:** 1 National Institute for Lasers, Plasma and Radiation Physics, 077125 Măgurele, Romania; lavinia.carpen@inflpr.ro (L.G.C.); veronica.satulu@infim.ro (V.S.); s_vizi@infim.ro (S.V.); bogdan.bita@inflpr.ro (B.I.B.); dinescug@infim.ro (G.D.); 2 Faculty of Physics, University of Bucharest, 077125 Măgurele, Romania; 3 National Institute of Materials Physics, 077125 Măgurele, Romania; elena.matei@infim.ro

**Keywords:** hybrid nanomaterials, plasma techniques, carbon nanowalls, tungsten oxides, nanoparticles, oxide sublimation, vapor transport, oxide deposition, fusion technology, sensors

## Abstract

We present hybrid nanomaterial architectures, consisting of carbon nanowalls (CNW) templates decorated with tungsten oxide nanoparticles, synthesized using a mechanism based on tungsten oxide sublimation, vapor transport, followed by vapor condensation, in the absence or presence of plasma. The key steps in the decoration mechanism are the sublimation of tungsten oxides, when are exposed in vacuum at high temperature (800 °C), and their redeposition on colder surfaces (400–600 °C). The morphology and chemical composition of the hybrid architectures, as obtained from Scanning Electron Microscopy and X-ray Photoelectron Spectroscopy, are discussed with respect to substrate nature and the physical conditions of synthesis. We pointed out that the decoration process is strongly dependent on the temperature of the CNW templates and plasma presence. Thus, the decoration process performed with plasma was effective for a wider range of template temperatures, in contrast with the decoration process performed without plasma. The results are useful for applications using the sensing and photochemical properties of tungsten oxides, and have also relevance for fusion technology, tungsten walls erosion and material redeposition being widely observed in fusion machines.

## 1. Introduction

The nanostructured carbon materials, namely carbon nanowalls (CNWs), are among the most studied materials. They are described as a network of interconnected two-dimensional carbon nanostructures (a few to tens of nanometers thickness and micrometer size lateral dimensions), oriented perpendicularly on the substrate [1]. Due to their extraordinary properties such as good electrical conductivity, chemical stability, large surface areas, high surface-to-volume ratio and sharp vertical edges [2,3,4,5] the CNW layers present many appealing applications. In particular, the open, porous architecture of the network of vertically oriented graphenic sheets, offering a large surface area for interfacing other nanomaterials, make CNW well suited to be used as templates for hybrid nano-architectures synthesis [6,7,8,9,10,11].

Promising functional hybrid nanostructures, characterized by novel properties, may be formed based on the decoration of tungsten (W) oxide on CNW templates. An important aspect, about combing these two materials, is highlighted by tungsten oxide unique properties, which makes it, nowadays, an important candidate for applications such as gas sensors [12] and, more recently, for photoelectrochemical water splitting [13,14]. On the other hand, tungsten oxides were studied with respect to their incidence in fusion technology, where they can form, sublimate, redeposit and affect the properties of the tungsten walls used in the fusion reactors [15].

Only a reduced number of articles regarding the application of CNWs as templates for the vertical distribution of tungsten or tungsten oxide nanocomposite fabrication have been reported to date. Thus, in [16] we reported the decoration of CNW template (obtained by Plasma enhanced Chemical Vapors Deposition—PECVD) with W nanoparticles (W NPs) obtained by magnetron sputtering combined with gas aggregation (MSGA) [17,18]. The decoration was performed by exposing the CNW substrates directly to the nanoparticles beam, in the MSGA setup. Moreover, in [19] is presented the synthesis of the WO_3_/CNW hybrid nanostructures using the hot filament chemical vapor deposition method; here the WO_3_ and CNW components are obtained in the same experimental setup.

Material deposition, via sublimation of solids and deposition of vapors, was previously applied for various materials, the examples including oxides [20], diamond [21] and polymers [22]. However, the investigation of plasma assisting of such processes is scarce; an example is the deposition of parylene [23]. Besides CNW template, deposition of nanostructured tungsten oxide via sublimation for gas sensing application was already proposed on porous silicon in [24,25]. WO_3_ nanowires were synthesized on porous silicon by thermal sublimation process using a horizontal furnace [26], similar to the one that is used in the present paper. The morphology of tungsten oxide nanowires was found to be influenced by the substrate temperature, more precisely on the substrate position in the furnace tube. Apart from experimental works, it is worth mentioning the theoretical methods [27,28] (such as ab-initio molecular dynamics, as well as other ab initio methods) which are available for addressing by atomistic simulation the structures of the resulting composite materials.

In the present work, we described the synthesis of the WO_3_/CNW hybrid structures using a mechanism based on tungsten oxide sublimation, vapor transport and vapor condensation, in the absence or presence of plasma, on CNW template. Herewith, the experimental setup is represented by a horizontal furnace tube that allows simultaneous heating and plasma ignition. The CNW substrates to be decorated and the source of the WO_3_ vapors were prepared in advance. Thus, the CNW templates were grown priory using PECVD method [29], while the WO_3_ source material was represented by metallic W nanoparticles, synthesized using MSGA. In order to form the tungsten oxide powder source, the metallic W nanoparticles were oxidized in the horizontal furnace, by a thermal process. The decoration process was performed in a typical experimental setup arrangement, placing the WO_3_ source in the center of the furnace hot zone (at 800 °C), while the CNW templates were placed in the extremity of the furnace hot zone, in positions corresponding to different temperatures (between 400–600 °C). The WO_3_/CNW hybrid nanostructures were investigated with respect to their morphology (using scanning electron microscopy—SEM) and chemical composition (by X ray photoelectron spectroscopy XPS). These results were used for inferring the influence of plasma over the decoration process.

## 2. Materials and Methods

### 2.1. Preparation of Tungsten Nanoparticles, Carbon Nanowalls and Their Hybrid Architectures

W nanoparticles and CNW layers were synthesized in advance using MSGA [17,18] and PECVD [29,30] method, respectively. Afterwards, the synthesis of CNW/WO_3_ hybrid nanostructures was performed using a horizontal furnace. The experimental setups and the conditions/parameters used for W nanoparticles, CNWs and for hybrid nanostructures synthesis are briefly described below.

#### 2.1.1. Synthesis of CNW Nanostructures

The CNWs synthesis was based on PECVD process. The reactor used for nanostructures synthesis consists of a vertically oriented, tubular stainless-steel chamber maintained under vacuum at a background pressure of 100 Pa. The expanding plasma source which sustained the PECVD process is mounted on the top side of the reactor chamber. Three gases (carrying gas: Ar, active gas: H_2_, and precursor gas: C_2_H_2_) are introduced into the chamber, at various stages of the process. Firstly, a RF (13.56 MHz) plasma discharge, expanding through a nozzle, is generated topside in Ar. The precursor and the active gas are supplied in expansion, in the nozzle vicinity, through an injection ring surrounding the plasma beam. The CNWs grow on a heated substrate, placed downstream the nozzle. These layers were obtained in Ar/H_2_/C_2_H_2_ mixtures (1050/25/2 sccm) at 300 W RF power. The substrate temperature was maintained at 700 °C and the distance from the injection ring to the substrate was around 5 cm. The deposition time for each sample was 60 min and the nanostructures were grown on silicon substrates. More details on the growth procedure have been presented elsewhere [29]. The obtained substrate contains large and well isolated layers of carbon (i.e., CNW), which are further used as templates for decoration with WO_3_.

#### 2.1.2. Synthesis of Tungsten Nanoparticles

The W nanoparticles were synthesized using MSGA method. A detailed description of the experimental setup and nanoparticle characterization can be found in [17,18]. The setup is formed from two chambers, an aggregation chamber (MSGA cluster source) which is in communication, through a 2 mm diameter aperture, with a vacuum deposition chamber. The cluster source consists of a water-cooled stainless-steel tube in which a magnetron sputtering plasma gun is mounted. In order to sustain the discharge, a radiofrequency generator (13.56 MHz, applied power P_RF_ = 80 W), provided with an impedance matching box, was used. The Ar mass flow rate is 5 sccm, corresponding to pressures of 80 Pa in the aggregation chamber and 0.5 Pa in the deposition chamber. The distance between the magnetron target (tungsten, 2 inches diameter, purity 99.95%) and the exit aperture defines the space where the aggregation of nanoparticles takes place (the length of this space was constantly maintained at 90 mm). The particles are formed in the cluster chamber and they are transported through the aperture in the deposition chamber, where particles are collected on microscope glass substrates. Using MSGA method, we obtained tungsten nanoparticles of about 100 nm in diameter, characterized by flower- like morphology (see SEM images in [17]). After synthesis, this nanoparticles powder was weighed and collected from the substrates on an alumina boat, in view of their afterwards oxidation in the linear furnace. In the following, the tungsten oxide material will be referred to as powder source.

#### 2.1.3. Synthesis of CNW/WO_3_ Hybrid Nanostructures

**Figure 1:**
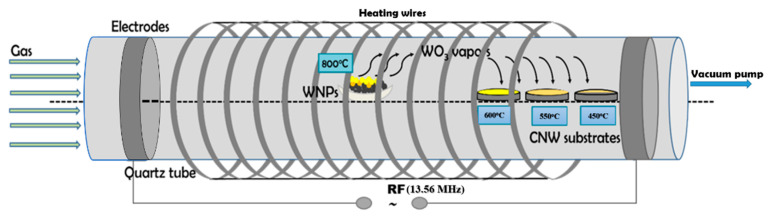
Schematic view of the experimental setup used for hybrid nanostructures synthesis in which the processes of heating which induced material sublimation, respectively thermal evaporation followed by condensation are highlighted.

The schematic view of the experimental setup used for nanoparticle oxidation and hybrid WO_3_/CNW nanostructures synthesis is presented in Figure 1. The processing chamber is part of a horizontal furnace (model CY-O1200-50ICS from ZHENGHZOU CY SCIENTIFIC INSTRUMENT CO) and consists of a quartz tube (external diameter 5 cm, length 60 cm) mounted inside a tubular oven. The quartz tube is connected with vacuum tight metal flanges and taps at the ends. The working gas is introduced through one end of the tube and the other end is connected to a preliminary vacuum pump (rotary oil pump), thus ensuring a continuous flow of the working gas through the quartz tube. The oven allows the heating of the samples inside the tube up to maximum 1100 °C. Additionally, the horizontal furnace was upgraded by us with two annular external electrodes (mounted on the quartz tube) for generating inside the tube a low temperature radiofrequency plasma.

**Figure 2:**
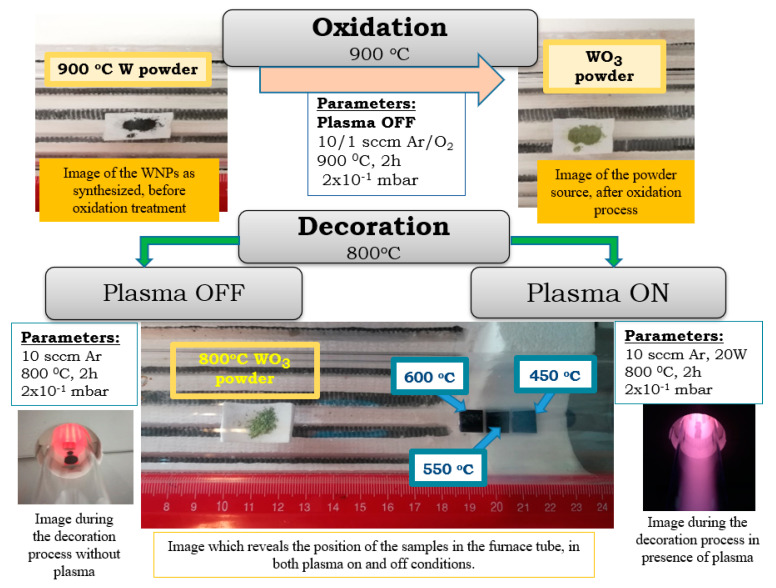
The schematic representation of the oxidation and decoration processes used for the hybrid nanostructures synthesis.

The WO_3_ source and the substrates were mounted as indicated in Figure 1. The heating temperature of the WO_3_ powder source was 800 °C. This value is located in the lower range of the WO_3_ sublimation domain (starting at 750 °C [26]) and it was chosen in order to have a moderate sublimation rate of the tungsten oxide. Other works, involving WO_3_ sublimation followed by condensation in linear furnaces, report heating temperatures from 700 °C [25] up to 1150 °C [24,26]. On the other hand, the substrates were placed in the extremity of the furnace tube, in positions corresponding to 600 °C, 550 °C, and 450 °C. By selecting these particular temperatures, the thermal degradation of the CNW substrates was avoided (around 700 °C in Ar [31]), the re-sublimation of the condensed WO_3_ was prevented and hybrid nanostructures synthesis was promoted, even in the case of the lower temperature limit. The hybrid nanostructures were synthesized based on tungsten nanoparticles, as primary tungsten material. The schematic representation of the processes used for hybrid nanostructures synthesis is presented in Figure 2. Therefore, for these nanostructures’ synthesis, firstly, the tungsten oxide powder source was obtained. This process will be described below as *OXIDATION*. Afterwards, hybrid nanostructures were obtained using two methods based on sublimation, vapour transport and condensation, which are characterized by *plasma on* or *plasma off* influence. This process will be presented as *DECORATION*.

The oxidation procedure. For each experiment, tungsten powder (40 mg) was loaded on an alumina boat and placed in the center of the quartz tube. After that, an Ar/O_2_ (gas ratio 10:1 total flow rate 11 sccm) gas mixture at a pressure of 25 Pa was established in the furnace. Then, the temperature in the tube furnace was increased gradually from room temperature to 900 °C, at a rate of 30 °C/min; after reaching the plateau temperature it was maintained for 2 h at the set temperature (900 °C). This temperature value was chosen for assuring rapid oxidation of WNPs [32], with the formation of a porous oxide with a large specific area prone to easily promote material sublimation. After treatment, the samples were then allowed to cool down back to ambient condition by natural convection, in the horizontal furnace.

The decoration procedure. The axial gradient of temperature that is present in the quartz tube is the basis for the decoration process. Thus, the oxidized powder was placed in the center of the furnace (where the temperature is 800 °C) while the substrates (three silicon substrates priorly coated with CNW layers) were placed in the colder extremity of the furnace tube. The substrates were placed at 8.5 cm, 9.5 and 10.5 cm away from the alumina boat in the downstream direction, points corresponding to local temperatures of 600 °C, 550 °C, and 450 °C. Following, the tube was pumped to a base pressure of 5.5 Pa, and pure argon (99.9999%) was introduced at a flow rate of 10 sccm (corresponding to a process pressure of 22 Pa). The decoration experiments were conducted via two instances, in the presence and absence of plasma (*plasma off*—first method, respectively *plasma on*—second method). After being maintained at the set temperature for 2 h, the furnace was freely cooled down to room temperature and the products were removed and analyzed as regarding their material properties. For selecting the decoration process parameters, we took into account that the initial CNW samples may be damaged by exposure to plasmas and oxygen, at high temperatures, for long periods of time [33].

### 2.2. Material Characterization

The morphology of the obtained products was analyzed by Scanning Electron Microscopy (SEM) measurements using a Gemini 500 equipment from Zeiss (Oberkochen, Germany). The chemical composition was determined by X-ray photoelectron spectroscopy (XPS, East Grinstead, UK), using a K-Alpha Thermo Scientific spectrometer (ESCALAB ™ XI +) equipped with a monochromatic X-ray source AlKα. Thus, the general spectra were registered, on the entire energy field, in order to determine the atomic composition for the investigated materials. The high-resolution spectra specific to the binding energies for elements of interest, namely, W4f, C1s, O1s and N1s, were recorded afterwards, in order to evaluate their binding states, as a result of the thermal processes combined with the plasma effects. Survey spectra were recorded at a pass energy of 50 eV, while the high-resolution spectra for specific binding energy regions were measured at a pass energy of 20 eV in order to evaluate the elemental bonding states of the as-resulted hybrid nanostructures. Peak positions were calibrated according to the standard position of C1s peak (284.8 eV). Both recording and processing, for low and high resolution XPS spectra, were done using advanced Avantage software (Thermo Avantage v5.976, Thermo Fisher Scientific, East Grinstead, UK).

## 3. Results and Discussion

As mentioned before, the preparation of hybrid nanostructures implies a mechanism based on oxide sublimation, transport and condensation. At high temperatures, the oxidized WNPs sublimate (at temperatures above 750 °C) [32] and vapors are released in the tube. The formed oxide vapors are transported by the gas flow and under the temperature gradient towards the substrates, where they condensate on the colder CNW surfaces.

Further on, experimental results obtained from oxidation and decoration processes are presented. Thus, as a result of the oxidation process, the powder source before and after the process is characterized. Subsequently, SEM images of the hybrid nanostructures obtained after CNW decoration are shown, pointing out the influence of plasma during the thermal treatments. For a better understanding of the process, at the same time, the changes that occur on carbon substrates will be presented, substrates that have been treated at the same conditions (800 °C, just pure Ar, for 2 h, in the absence and presence of plasma). The chemical properties of the presented materials are inferred from XPS measurements.

### 3.1. Properties of the Tungsten Oxide Particles

**Figure 3:**
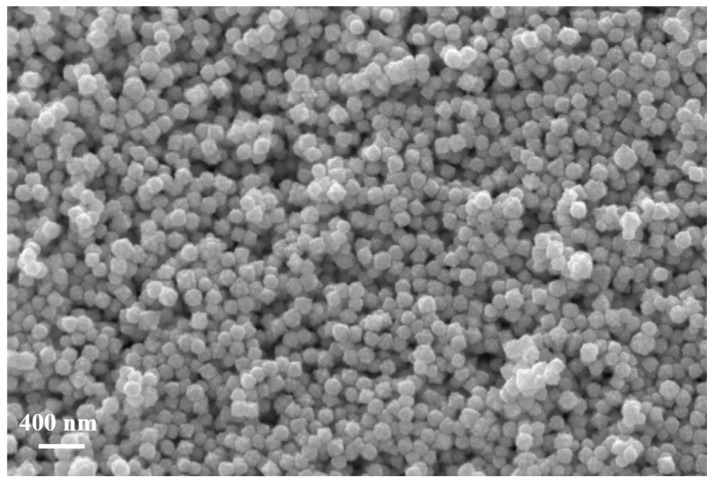
Initial tungsten nanoparticles. after synthesis using MSGA method.

**Figure 4:**
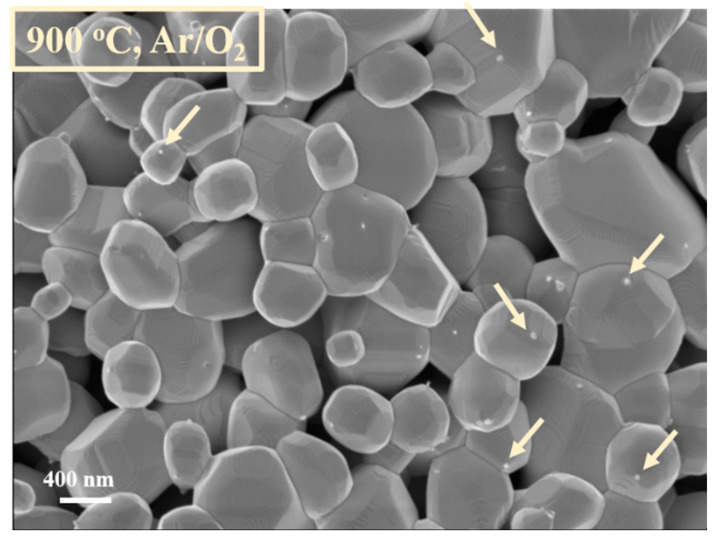
Tungsten oxide particles, obtained after the oxidation process.

Within the oxidation, a small amount of oxygen (1 sccm oxygen) was mixed with Ar (10 sccm) into the quartz tube, in order to help the tungsten nanoparticles oxidation. The treatment was performed without plasma, at a temperature of 900 °C, at a pressure of. 25 Pa. Important color aspects regarding the power source were visible before and after oxidation (Figure 2). Thus, before the oxidation process, the powder source was black. After the oxidation process, when a small amount of oxygen was used, the powder turned yellow (Figure 2). This color change indicates a modification in the oxidation state of the nanoparticles [32] Moreover, during the oxidation process, the powder mass increases by approximately 10 percent. Regarding morphological aspects, during the oxidation process, the tungsten nanoparticles tend to lose their nano-scale dimension (Figure 3), fusing in crystalline micro sized particles (Figure 4). The oxidation of the WNPs is favored by the diffusion of oxygen along the paths defined by the facets of the nanocrystallites present in the structure of the flower like nanoparticles [17] and by the possible crystalline defects [34]. Apart from the oxidation effect, further heating of the sample (up to 900 °C) leads to the recrystallization of the oxidized particles and the fusion of particles that are in contact. These effects are similar to those observed during the annealing of nanostructured thin films of WO_3_ [35]. As the temperature in the furnace reaches 750 °C [26], evaporation of the WO_3_ starts and an Ostwald ripening process (i.e., evaporation of smaller particles and redeposition of the vapors on the bigger ones) is possible to happen. This supposition is supported by the presence of small particles (20–50 nm, marked with arrows in Figure 4) on the surfaces of the bigger ones, similar to those presented in [36] for C particles.

**Figure 5:**
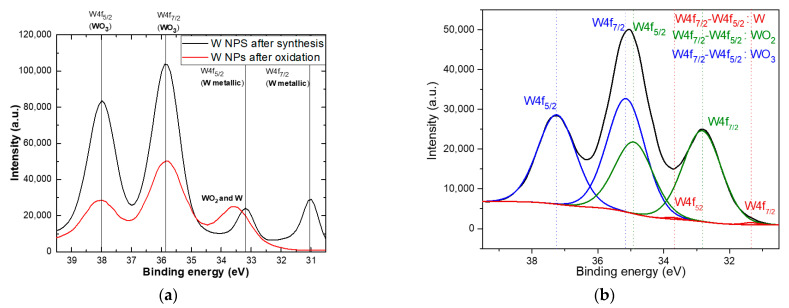
(**a**) Comparative XPS spectra of initial tungsten nanoparticles, respectively tungsten oxide particles, obtained after oxidation process. (**b**) XPS deconvolution results of the W4f region for W nanoparticles obtained after oxidation.

In order to investigate the difference between chemical composition and bonding state for as-synthesized particles and the particles after oxidation, XPS measurements were presented in Figure 5a. With a black line, the XPS spectrum of the as-synthesized nanoparticles is presented. This spectrum shows a mixture of metallic tungsten and tungsten oxides, because tungsten is well known to oxidize quickly in the ambient atmosphere [17,37,38]. After thermal treatment at 900 °C in a mixture of Argon and Oxygen atmosphere, it can be noticed a significant transformation of metallic tungsten in tungsten oxide. To distinguish between the different states of oxidation, in Figure 5b, the deconvolution of W4f region is presented. W4f peak area was defined by performing an appropriate background correction, namely, Shirley type background subtraction, across the binding energy range, excluding the W3p peak and taking into account the sensitivity factor automatically employed by the instrument. The W4f spectrum was fitted with Gaussian Lorentz Product (GLP) line shape, best fits of this peak being obtained with GLP and a very low value of residual fit, where the energy splitting (2.18 eV) and full width at half maximum (FWHM) fitting parameters were initially constrained and then allowed to vary. The presence of the WO_3_ doublet is observed at 35.14 eV (W4f7/2) and 37.25 eV (W4f5/2) with a contribution of 54.88%. The WO_2_ doublet is observed at 32.83 eV (W4f7/2) and 34.90 eV (W4f5/2) with a contribution of 44.74% [17,39,40]. W metallic doublet is still present after oxidation at 31.35 eV (W4f7/2) and 33.72 eV (W4f5/2), but with a small contribution of just 0.39%. Otherwise, the low contribution of W metallic bonds highlighted the successful oxidation that have place after the thermal treatment.

### 3.2. Characterization of the Hybrid Nanostructures Synthesized in the Absence of Plasma (PLASMA-OFF)

#### 3.2.1. Morphological Aspects

**Figure 6:**
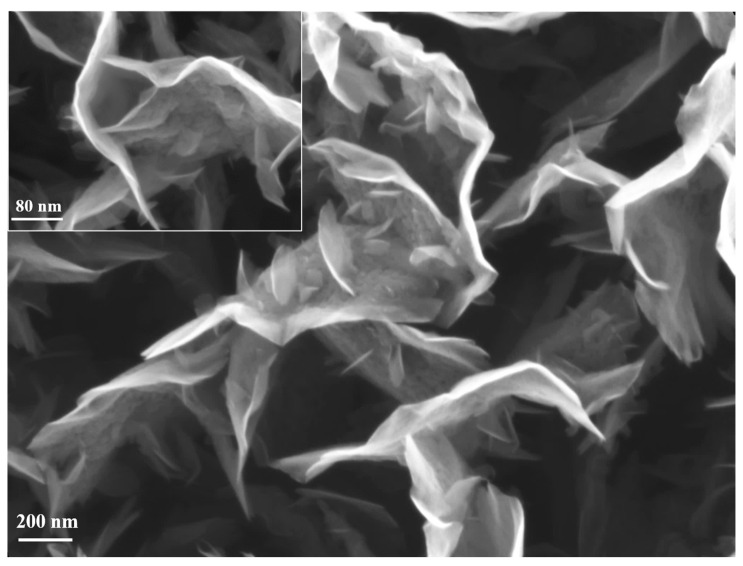
CNW control sample, after synthesis using PECVD method.

**Figure 7:**
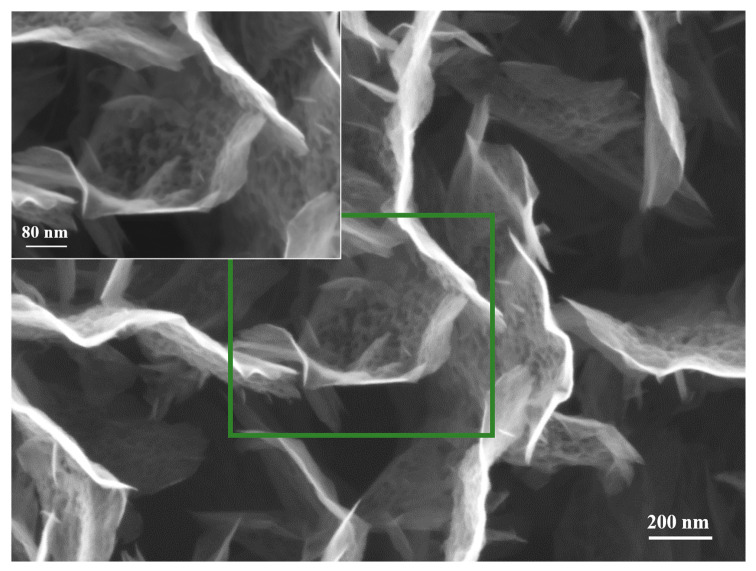
CNW after thermal treatment (No powder source) (600 degrees, plasma off).

First of all, the CNW templates proved to be very resistant/durable when are exposed to high temperatures [31]. This aspect was highlighted also by our previous results, in which we have shown, by thermogravimetry measurements, that CNW templates are thermally stable in Ar atmosphere up to 712 °C [31]. Compared to the as-synthesized CNW sample, denoted by us as “control sample” (Figure 6), only slight changes in morphology (Figure 7) may be observed for the heated samples, and only on the substrate positioned in the furnace tube at a higher temperature of 600 °C. Apparently, the sheets become more porous after heating (aspect highlighted by comparing the insets that were added in Figure 6, respectively Figure 7). The samples exposed at lower temperatures did not show changes compared to the control sample.

**Figure 8:**
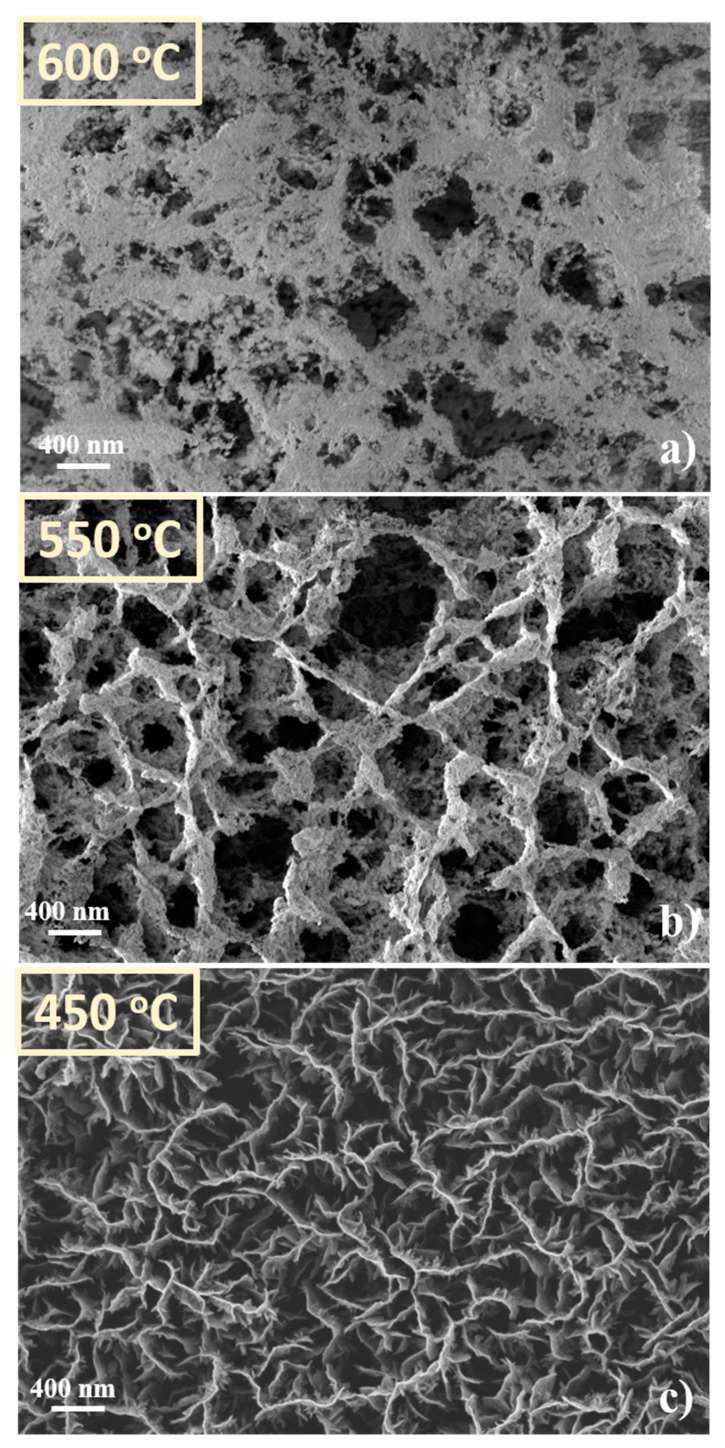
SEM images of CNW surface obtained after WO_3_ decoration. Decoration obtained with *plasma off* at different temperatures (**a**) 600 °C; (**b**) 550 °C; (**c**) 450 °C; corresponding with the positions of the substrate in the furnace.

Concerning the decoration process, it was proved to be very dependent on the substrate position in the furnace. This aspect is illustrated by the series of SEM images presented in Figure 8. At 600 °C (Figure 8a), a discontinuous layer of small size particles is observed on the sample, which apparently did not respect the CNW template features. Contrary, at 450 °C (Figure 8c), is hard to observe any decoration of the CNWs. The decoration process leads to a hybrid architecture around 550 °C (Figure 8b), where a porous structure with clear dispersion of particles on the CNW skeleton can be observed.

#### 3.2.2. Chemical Composition

**Figure 9:**
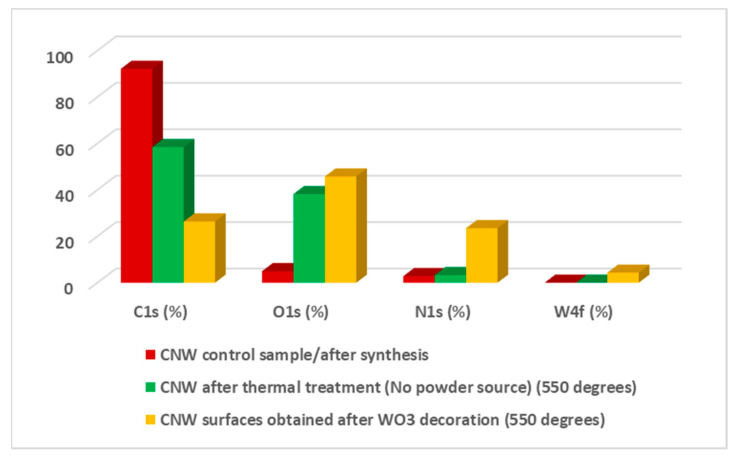
Bar diagram of the elemental composition corresponding with the four major zones (C1s, O1s, N1s, W4f) for all three investigated samples (CNW control sample, CNW after thermal treatments, respectively nanostructures obtained after WO_3_ decoration) in *plasma off* condition.

In order to highlight the chemical composition of the nanostructures obtained in plasma OFF conditions, XPS measurements were performed. Below, we present the investigations performed on the samples at 550 °C, as representing the middle temperature. These measurements emphasized the difference regarding the chemical composition and bonding state between CNW treated sample (without powder source) and hybrid nanostructures (obtained using the powder source in the center of the tube), both obtained in *plasma off* conditions. The atomic concentrations of four major zones (O1s, C1s, N1s, W4f) are presented Table 1. In this table, the difference between CNW control sample (after PECVD growth), respectively the CNW surface after thermal treatment (just Ar, 2 h treatment, no powder source), in contrast with the CNW surfaces obtained after tungsten oxide decoration is presented. This table is completed with a bar diagram, presented in Figure 9, in which the modifications regarding atomic concentrations, after each treatment, are presented. It can be observed a decreasing of carbon component (from 92.19%, the percent obtained for the as-synthesized samples, up to 58.48%, obtained for CNWs thermal treated samples, respectively 26.39% obtained for hybrid nanostructures obtained in plasma OFF condition) Therefore, the maximum concentration of C1s is obtained for the control sample and this concentration decrease to a minimum value, when the tungsten oxide powder source is used in the center of the quartz tube, in order to obtain the hybrid nanostructures. In that case, C1s is replaced with a higher amount of O1s and N1s and a small amount of W4f. Moreover, the carbon amount decreased after thermal treatment (from 92.19% up to 58.48%), which can reveal the CNW deterioration. Table 1 emphasizes the important contribution of atomic oxygen in the as- obtained carbon materials, both with and without the powder source. The atomic oxygen contribution can be suggested, firstly, by the affinity of this element for metals (tungsten in our case), and secondly, by the surface oxidation that occurs in the ambient atmosphere [17,37,38]. The presence of N1s can be correlated with atmospheric air impurities that may be present in the quartz tube, due to the low vacuum (about 10 Pa) used during the decoration processes. When the source material was used in the center of the tube (the powder source which consists of a mixture of WO_2_ and WO_3_, how it was presented in Figure 5b), in the as- formed hybrid material the presence of W4f is highlighted, but together with a non-negligible amount of N1s and O1s, which may indicate the presence of some bonding states between these elements.

**Figure 10:**
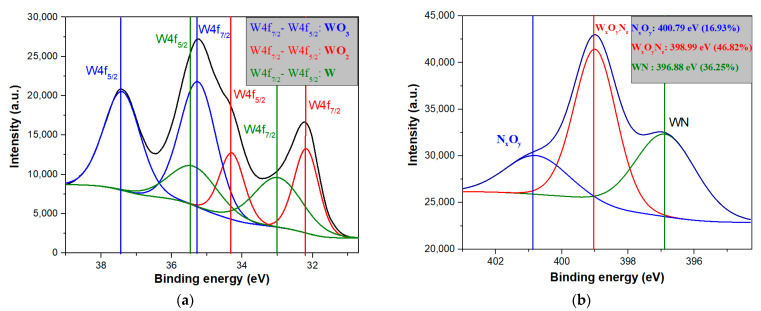
XPS deconvolution results for hybrid nanostructures obtained in *plasma off* condition in (**a**) W4f region (**b**) N1s region.

Therefore, in order to have a better understanding of the elemental bonding states for the hybrid nanostructures (CNW surface decorated with tungsten oxide nanoparticles) obtained in plasma OFF conditions, high resolution spectra have been recorded for W4f, O1s, respectively N1s binding energy regions. In Figure 10 the deconvolutions, corresponding to W4f (Figure 10a) and N1s (Figure 10b), are presented. The presence of the WO_3_ doublet is observed at 35.25 eV (W4f7/2) and 37.41 eV(W4f5/2) with a contribution of 50.51%. The WO_2_ doublet is observed at 32.18 eV (W4f7/2) and 34.29 eV(W4f5/2) with a contribution of 22.95% [17,39,40]. Moreover, due to the higher atomic percentage of nitrogen, the WN chemical doublet is present at 32.99 eV(W4f7/2) and 35.36 eV(W4f5/2) [41], with a contribution of 26.54%. To highlight the presence of WN chemical bond, the deconvolution corresponding to N1s is presented in Figure 10b. Therefore, the WN chemical bond is observed at 396.88 eV with a contribution of 36.25%. Moreover, W_x_O_y_N_z_ and N_x_O_y_ are presented at 398.99, respectively 400.79 [42], with a contribution of 46.82, respectively 16.93%.

**Figure 11:**
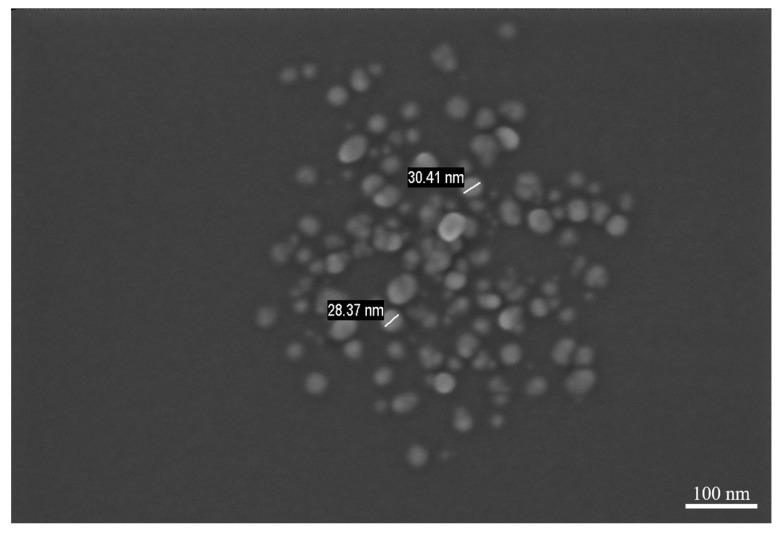
Image of nanoparticles obtained after the condensation process, on a clean silicon substrate, without CNW template, at 550 °C.

Despite the results presented, we still question whether CNWs template influence this decoration process, implying whether decoration occurs on another type of substrate. Therefore, a similar experiment was done in which in the center of the quartz tube the powder source was positioned, but, in the extremity of the tube, just clean silicon substrates were used, in the same positions as before (i.e., for those used for CNW substrates). Figure 11 present the SEM image of such a Si substrate after the decoration process. In this particular case, even if the nanoparticles are still observed on the substrate, these nanoparticles are not uniform on the entire surface, how we obtained within the hybrid architectures. Nanoparticles, which are decorating the Si substrate, are characterized by a very low diameter, approximately 30 nm and they are covering only some zones of the surface. These aspects are presented in Figure 11. Therefore, it is important to be mentioned that without CNW templates, a reduced number of nanoparticles are obtained and the distributions on the surface lose their uniform covering. This result is related to the number of condensation centers existing on the two different substrates. A larger number of condensation centers (edges, defects, linked polar groups, etc.) are existing on the easy to be activated carbon surface in comparison with silicon substrates.

### 3.3. Characterization of the Hybrid Nanostructures Synthesized in Presence of Plasma (PLASMA-ON)

#### 3.3.1. Morphological Aspects

**Figure 12:**
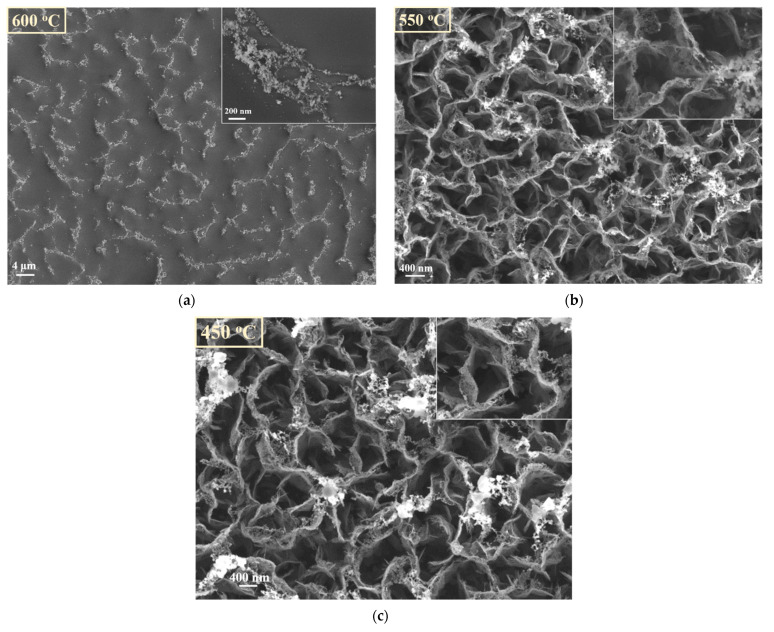
CNW template after thermal treatment in presence of plasma, at different temperatures (**a**)—the first position corresponding at 600 °C; (**b**)—the second position corresponding at 550 °C; (**c**)—the third position corresponding at 450 °C).

In Figure 12 are presented the SEM images of the CNW samples treated in absence of the source material (W oxide) at 600 °C, 550 °C and 450 °C, respectively. In presence of plasma, in particular at high temperatures, the CNWs substrates were very affected. The CNW nanostructures are practically destroyed at 600 °C. Only traces of carbon material are remaining along the contours of the initial positions of CNW, organized as foam clouds (inset in Figure 12a). The layers are less affected at 550 °C and 450 °C; still, compared with Figure 6 (i.e., the SEM image of the control sample), the erosion signs are clearly observed (more details about the surfaces are presented in the insets added in Figure 12b, respectively Figure 12c). The edges of CNW sheets are ragged; foam clouds are observed again on the top of the layers of carbon, suggesting a possible transformation of the top edges of CNWs in clouds of carbon nanoparticles.

**Figure 13:**
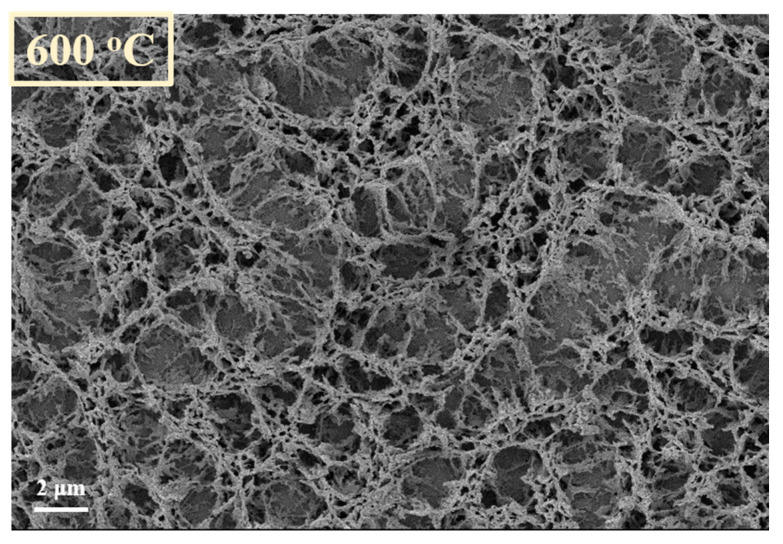
Image of hybrid nanostructures obtained after the decoration process, in presence of plasma, at 600 °C.

**Figure 14:**
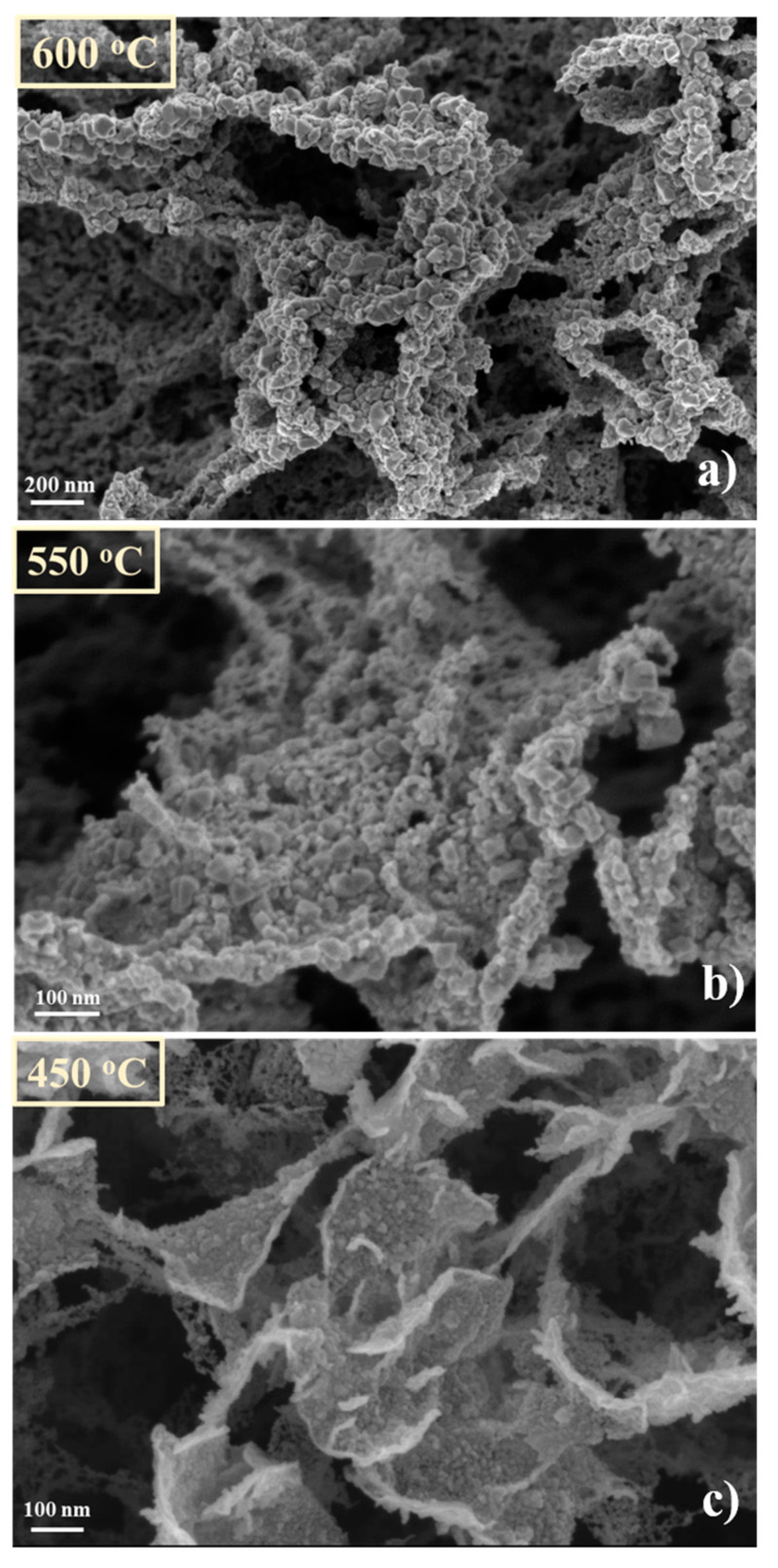
SEM images of CNW surfaces after decoration process performed in presence of plasma (*plasma on*), at different temperatures (**a**)—the first position corresponding at 600 °C, (**b**)—the second position corresponding at 550 °C, (**c**)—the third positions corresponding at 450 °C).

In Figure 13 is presented the SEM image of a decorated sample obtained in *plasma on* condition at 600 °C (corresponding with Figure 14a, but at a different magnification). It is interesting to observe that hybrid architecture, with a morphology similar to CNW layers, is obtained. This observation indicates, as a result of a direct comparison with Figure 12a, that in presence of the source material, the destroying of CNW template is diminished.

Thus, when the decoration occurs, grains are deposited on CNW template and this template remains intact, even if the temperature is high. In contrast with the results obtained without plasma, the CNW decoration process performed in Ar plasma was effective for all working temperatures. These aspects are highlighted in Figure 14, where SEM images of the decorated samples at 600 °C, 550 °C, 450 °C are shown. In this situation, the oxide nanoparticles totally cover the CNW walls. The size of the decoration grains increases with the substrate temperature. In addition, these images suggest that the amount of tungsten nanomaterial on the surface increases with temperature, being evenly distributed on the sheet’s surfaces. So, we conclude that, using plasma, the hybrid nanostructures are successfully obtained in a wider range of parameter values, comparing to the decoration obtained in absence of plasma. This result can be explained as an effect of the additional energy brought by the discharge and by the ions bombardment which, on the one side, enhance the sublimation rate in the source zone and, by the other side, increase the number of condensation centers in the deposition zone, thus favoring a fast and deep deposition of an oxide layer on the overall carbon template. This conformal oxide layer prevents a further contact of the carbon material with the eroding plasma, playing thus the protective role of the template. This protective effect explains the hybrid architecture formation at the temperature where normally the template is destroyed in plasma (600 °C). The activation and the larger amount of released vapors explain why such architectures are obtained at low temperatures like 450 °C.

#### 3.3.2. Chemical Composition

**Figure 15:**
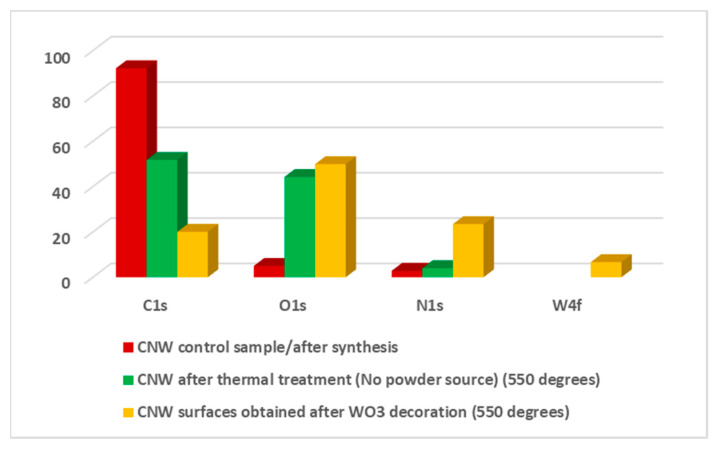
Bar diagram of the elemental composition corresponding with the four major zones (C1s, O1s, N1s, W4f) for all three investigated samples (CNW control sample, CNW after thermal treatments, respectively nanostructures obtained after WO_3_ decoration) in *plasma on* condition.

XPS measurements were performed in order to highlight the chemical composition of the nanostructures obtained in *plasma on* conditions. As in the case of *plasma off*, we will present the results obtained at 550 °C. Therefore, we made a comparison between the CNW control sample, after synthesis using PECVD, the CNW thermally treated sample in the presence of plasma (without the powder source), respectively the hybrid nanostructures obtained after tungsten oxide decoration (with powder source in the center of the quartz tube). The atomic concentrations of the elements (O1s, C1s, N1s, W4f) are presented in Table 2. Regarding the CNW samples, before and after thermal treatment, when plasma was used, the C1s percent decreases from 92.19% up to 51,78%. However, between these two samples, the O1s percent shows a significant increase in the case of the treated sample (from 4.99% observed in the control sample, up to 44.21%, the atomic percentage obtained for the CNW sample after the thermal treatment, without powder source). The N1s presents a small increase of approximately two percent, after thermal treatment. The decrease in C1s concentration is obvious for the hybrid nanostructures, obtained after WO_3_ decoration (from 92.19%, obtained for the as-synthesized CNW sample, up to 20.05%). Moreover, for the same sample, the decoration with tungsten element is observed by the presence of W4f in the total atomic concentration, with a percent of 6.65%. Directly connected with the metallic part, an important part of the total concentration is represented by O1s [17,37,38], with a percentage of 49.87%. Besides C1, W4f, and O1s a large percentage of N1s of 23.43% is highlighted. All these aspects are presented in the diagram bar from Figure 15.

**Figure 16:**
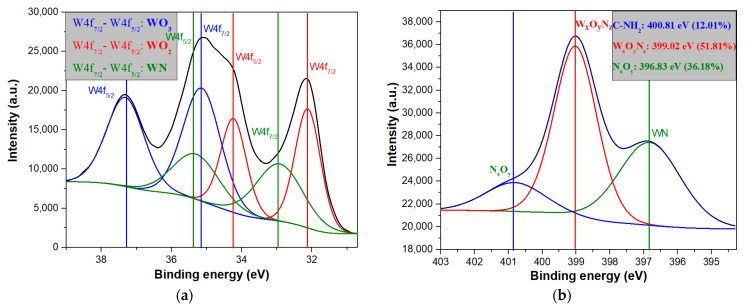
XPS deconvolution results for hybrid nanostructures obtained in *plasma on* condition in (**a**) W4f region (**b**) N1s region.

For better knowledge of W-O-N type bonds presence in the synthesized nanostructures, in *plasma on* conditions, in the following, we will discuss the deconvolution of the specific regions of interest. Therefore, in Figure 16a is presented the deconvolution of W4f, while in Figure 16b the deconvolution for N1s specific region is presented. Regarding the W4f region, the WO_2_ doublet is observed at 32.12 eV (W4f7/2) and 34.23 eV (W4f5/2) with a contribution of 30.54%. The presence of the WO_3_ doublet is observed at 35.14 eV (W4f7/2) and 37.32 eV (W4f5/2) with a contribution of 42.68% [17,39,40]. Moreover, due to the higher atomic percentage of nitrogen, the WN chemical doublet is present at 32.93 eV (W4f7/2) and 35.3 eV (W4f5/2), with a contribution of 26.78% [41]. In Figure 16b, the WN chemical bond is observed at 396.83 eV with a contribution of 36.18%. Moreover, W_x_O_y_N_z_ and N_x_O_y_ are presented at 399.02, respectively 400.84 [42], with a contribution of 51.81%, respectively 12.01%.

## 4. Conclusions

We demonstrated a new route for obtaining hybrid nano-architectures consisting of tungsten oxide nanoparticles conformally distributed onto a network of carbon nano-walls. The tungsten oxide-carbon hybrid nanomaterial was obtained through a sequence of processes consisting of oxidation of tungsten nanoparticles followed by oxide sublimation, vapor transport and condensation on a nano-carbon template. Experiments were performed with and without plasma assistance.

During the oxidation process, the tungsten nano-particles lose the nanometric scale, and transform into a micron-size powder of WO_3_ and WO_2_ oxides. When the oxide powder was subjected to sublimation at high temperature of 800 °C, it acted as a source of vapors which successfully lead, both in *plasma off* and *plasma on* conditions, to formation of hybrid architectures by condensation on colder (range 450 °C to 600 °C) CNW substrates.

We showed that the decoration process depends strongly on the substrate temperature and plasma presence. While the CNW template alone (without vapors) proved to be only slightly affected by exposure to high temperature, it is significantly degraded at high temperature in plasma presence. When adding the source of vapors this degradation is strongly reduced, revealing that the condensed oxide plays a protective role for the template. Besides, we were able to obtain a uniform decoration in the whole range of the used substrate temperature values, with nanoscale oxide particles, on the entire surface of the vertical carbon sheets, in contrast with the experiments performed in absence of plasma, when an efficient decoration was obtained only at 550 °C. This result was assigned to the sublimation enhancement and activation of carbon template with condensing centers, by ions bombardment.

The described method leads to hybrid nanostructures that can certainly find application in photoelectrochemical decomposition of water as a renewable energy source [13,14], respectively in gas sensor applications [12,43]. Moreover, the oxidation, vapor transport and condensation processes, evidenced in this paper, may contribute to the understanding of erosion and redeposition phenomena occurring in the fusion machines equipped with tungsten walls [15].

## Figures and Tables

**Table 1:** The atomic concentration of the obtained hybrid nanostructures in *plasma off* conditions.

	C1s (%)	O1s (%)	N1s (%)	W4f (%)
CNW control sample/after synthesis	92.19	4.99	2.83	-
CNW after thermal treatment (No powder source) (550 °C)	58.48	38.2	3.32	-
CNW surfaces obtained after WO_3_ decoration (550 °C)	26.39	45.77	23.51	4.33

**Table 2:** The atomic concentration of the obtained hybrid nanostructures in *plasma on* conditions.

	C1s (%)	O1s (%)	N1s (%)	W4f (%)
CNW control sample/after synthesis	92.19	4.99	2.83	
CNW after thermal treatment (No powder source) (550 °C)	51.78	44.21	4.01	
CNW surfaces obtained after WO_3_ decoration (550 °C)	20.05	49.87	23.43	6.65

## Data Availability

The data that supports the findings of this study are available within the article. Any additional data relevant to this study are available from the author upon reasonable request.
